# IFI16 is involved in HBV-associated acute-on-chronic liver failure inflammation

**DOI:** 10.1186/s12876-018-0791-1

**Published:** 2018-05-09

**Authors:** Xiuqing Pang, Xinhua Li, Zhishuo Mo, Jing Huang, Hong Deng, Ziying Lei, Xingrong Zheng, Zhiying Feng, Dongying Xie, Zhiliang Gao

**Affiliations:** 1 Department of Infectious Diseases, Third Affiliated Hospital of Sun-Yat-SenUniversity, GuangZhou, China; 2 GuangDong Provincial Key Laboratory of Liver Disease, Third Affiliated Hospital of Sun-Yat-Sen University, GuangZhou, China; 3 Department of Pathology, Third Affiliated Hospital of Sun-Yat-Sen University, GuangZhou, China; 4 GuangZhou, China

**Keywords:** IFN inducible protein 16 (IFI16), Chronic hepatitis B (CHB), HBV-associated acute-on-chronic liver failure (HBV-ACLF), Inflammation

## Abstract

**Background:**

Hepatitis B virus (HBV) is a hepatotropic DNA virus, and its DNA may be a potent inflammatory molecule. Interferon-inducible protein 16 (IFI16), a newly discovered DNA sensor, plays an important role in the process of inflammation in viral infections. Our study sought to identify a correlation between IFI16 expression and inflammation in patients with chronic hepatitis B (CHB) and HBV-associated acute-on-chronic liver failure (HBV-ACLF).

**Methods:**

We performed flow cytometry to measure IFI16 levels in peripheral blood mononuclear cells (PBMC) and used immunohistochemistry and western blotting to measure IFI16 protein levels in liver tissues. The cellular source of IFI16 was detected using double immunofluorescence. All datum were analyzed using SPSS 13.0 and GraphPad Prism 6.

**Results:**

The number of IFI16+ cells was significantly associated with the degree of inflammation. In detail, the number of IFI16+ cells was higher in livers but lower in PBMCs in HBV-ACLF patients than those in CHB patients and healthy controls. There was no significant difference between CHB patients and healthy controls in numbers of IFI6+ cells in livers and PBMCs. There was no significant relationship between IFI16 expression levels and HBV parameters. Furthermore, IFI16 was expressed in the nucleus of Kupffer cells (KCs), endothelial cells, natural killer cells, dendritic cells, and hepatic stellate cells in healthy donors and CHB patients, but only in the cytoplasm of KCs in the livers of HBV-ACLF patients.

**Conclusions:**

IFI16 was closely related to the degree of inflammation in CHB and HBV-ACLF patients and may serve as a vital contributor to the pathogeneses of liver damage in HBV-ACLF.

## Background

Hepatitis B virus (HBV) is a non-cytopathic human Hepadnavirus that causes a spectrum of diseases, such as chronic hepatitis B (CHB), liver cirrhosis, primary hepatocellular carcinoma, and liver failure. The pathology of HBV disease is closely associated with chronic inflammation, a dynamic process orchestrated by the complex interplay between virus replication and host immune response. It is widely accepted that adaptive immune responses play major roles in HBV infection [1]; however, the role of innate immunity during HBV infection is not to be well understood. The innate immune system is activated by viruses through pattern-recognition receptors (PRRs) that recognize specific viral structures, such as viral protein, single-stranded RNA and double-stranded RNA. Following recognition, recruitment of distinct adaptor proteins will initiate sequentially signaling cascades to induce inflammation cytokine production in virus-infected cells. Several PRRs, including Toll-like receptors (TLRs), RIG-I-like receptors (RLRs), and NOD-like receptors (NLRs), have been found to play important roles in HBV sensing and the formation of inflammasomes in the liver [2]. Current evidence suggests that a new type of PRRs, named DNA sensors, can detect microbial DNAs and initiate innate responses to provoke inflammation. HBV is a hepatotropic DNA virus, and thus, its DNA may be a potent immune stimulatory molecule.

Interferon inducible protein 16 (IFI16), a newly discovered molecule that senses DNA in cells and stimulates stimulator of IFN genes (STING)-dependent IFN expression and apoptosis-associated speck-like protein containing caspase recruitment domain (ASC)-dependent inflammation activation [3]. Based on its structure, IFI16 is clustered into the PYHIN family of proteins which is defined by one or two C-terminal DNA binding HIN domains and an N-terminal homotypic protein–protein interactions pyrin domain (PYD). Currently, four members of the PYHIN family have been discovered: absent in melanoma 2 (AIM-2), interferon-inducible protein X (IFIX), Myeloid cell nuclear differentiation antigen (MNDA) and IFI16. Previous studies showed that AIM2 can stimulate inflammasome activation and downstream cleavage of pro-interleukin (IL)1b and pro-IL18 to bioactive cytokines via interactions between the PYD and the inflammasome adaptor protein ASC [4]. Such AIM2-ASC inflammatory signaling pathways activation may occur during HBV infection [5]. To date, IFI16 is the only one member of this family with two HIN domains, termed HIN-A and HIN-B [6], and these two domains potentially increase the DNA-binding ability. Recently, IFI16 was reported to also have the capacity to activate ASC-dependent inflammasomes during HSV-1, Kaposi sarcoma-associated herpes virus (KSHV) or Epstein–Barr virus infection [7–9]. Furthermore, IFI16 was demonstrated to stimulate IFN expression through a mechanism through STING, TANK-binding kinase 1 (TBK1), and interferon regulatory factor-3 (IRF-3) signaling in a DNA-driven manner. Based on its unique structure of HIN domains, IFI16 can bind different nucleotide structures, such as dsDNA, superhelical and cruciform DNA, HIV-Nef DNA. Studies have demonstrated the induction of IFN responses during infection with herpes simplex virus (HSV), Human cytomegalovirus (HCMV), HIV and lentiviruses, as well as with *Listeria monocytogenes* [10–13].

Taken together, IFI16 plays an important role in the process of immune response in viral infections. Therefore, we predicted that IFI16 may play a vital role in the development of HBV sensing and inflammasomes in the liver, although the expression of IFI16 in human HBV infection has not been thoroughly investigated. During HBV infection, peripheral blood mononuclear cells (PBMCs) are very important part of the peripheral immune system. In addition, the liver is well-known to be an immunological organ with a predominant innate immune system. Our laboratory investigated whether IFI16 is expressed in PBMCs and livers in CHB and HBV-ACLF patients and whether HBV DNA is recognized by IFI16.In addition, we examined the correlation between IFI16 expression and the inflammation.

## Methods

### Patients

All patient samples were collected in our hospital from June 2013 to December 2014. Peripheral blood samples were collected from 31 CHB patients and 13 patients with HBV-associated acute-on-chronic liver failure (HBV-ACLF); 16 sex-matched healthy individuals were enrolled as normal controls. Liver tissue samples were collected from 59 CHB patients who had liver biopsies, 17 HBV-ACLF patients who received a liver transplant, and 19 healthy liver transplant donors. CHB patients were defined as those who were HBsAg-positive for more than 6 months and that exhibited symptoms or signs of hepatitis and abnormal hepatic function on occasion or those that had the disease histologically confirmed. By contrast, the diagnosis of HBV-ACLF was based on clinical evidence of either ≥grade 2 hepatic encephalopathy, abrupt and obvious increase in ascites, spontaneous bacterial peritonitis, or hepatorenal syndrome; these clinical criteria were associated with recent development of jaundice [total bilirubin [TBIL] ≥10.0 mg/d or rapidly rising levels of TBIL(TBIL≥1.0 mg/dL/day)] and a prothrombin activity ≤40%. Patients with autoimmune liver diseases or cancer were excluded. All patients were seronegative for markers of hepatitis A, C, D, and E viruses and for HIV virus. The model for end-stage liver disease (MELD) score [14] was used to assess disease severity and prognosis. Alanine aminotransferase (ALT) and aspartate aminotransferase (AST)levels, bilirubin levels, international normalized ratio (INR), creatinine, HBV DNA load, and MELD score were measured. The study protocol was approved by the ethics committee of our hospital and written informed consent was obtained from each subject. The basic characteristics of the enrolled subjects are listed in Tables 1 and 2.

**Table 1:** Clinical characteristics of the populations with PBMCs enrolled in the study

group	NC	CHB	HBV-ACLF
case	16	31	13
sex(M/F)	12/4	25/6	12/1
age(years)	29.5(26-63)	36(21-61)	44(37-67)
ALT(U/L)	21.5(11-34)	50(11-217)	90(46-245)
AST(U/L)	22(12-34)	46(21-222)	117(58-158)
Tbil(umol/L)	ND	12.6(4.0-119.9)	473.8(51.9-720)
INR	ND	1.04(0.88-1.23)	2.87(2.01-4.68)
PTA(%)	ND	94(72-128)	26(16-39)
Log10 (HBV DNA)	ND	5.17(1.30-8.23)	3.25(1.51-8.19)
HBsAg-positive	0	31	13
HBsAb-positive	16	0	0
HBeAg-positive	0	13	3
HBeAb-positive	0	18	10
HBcA-bpositive	0	31	13
MELD score	ND	ND	28.2±1.22

Data are shown as median and range

ACLF, acute on chronic liver failure; CHB, chronic hepatitis B; ALT, alanine aminotransferase; AST, aspartate aminotransferase; Tbil, total bilirubin;HBcAb, hepatitis B c antibody; HBeAb, hepatitis B e antibody; HBeAg, hepatitis B e antigen; HBsAb, hepatitis B s antibody; HBsAg,hepatitis B s antigen; HBV, hepatitis B virus; ND, not determined; NC,normal control; PTA, prothrombin time activity; MELD Model for end-stage liver disease

**Table 2:** Clinical characteristics of the populations with liver tissues enrolled in the study

group	NC	CHB	HBV-ACLF
case	19	59	17
sex(M/F)	ND	46/13	16/1
age(years)	ND	37(23-65)	41(29-61)
ALT(U/L)	ND	50(7-570)	40(11-156)
AST(U/L)	ND	37(15-256)	92(40-202)
Tbil(umol/L)	ND	12.1(2.6-119.9)	600.6(238-809)
INR	ND	1.03(0.87-1.35)	2.88(2.11-5.83)
PTA(%)	ND	95(63-128)	21(11-35)
Log10 (HBV DNA)	ND	5.42(0-8.54)	2(0.83-8.05)
HBsAg-positive	ND	59	17
HBsAb-positive	ND	0	0
HBeAg-positive	ND	24	2
HBeAb-positive	ND	35	15
HBcA-bpositive	ND	59	17
MELD score	ND	ND	33.33±2.02

Data are shown as median and range

ACLF, acute on chronic liver failure; CHB, chronic hepatitis B; ALT, alanine aminotransferase;AST, aspartate aminotransferase; Tbil, total bilirubin; HBcAb, hepatitis B c antibody; HBeAb, hepatitis B e antibody;HBeAg, hepatitis B e antigen; HBsAb, hepatitis B s antibody; HBsAg, hepatitis B s antigen; HBV, hepatitis B virus; ND, not determined; NC, normal control; PTA, prothrombin time activity. MELD Model for end-stage liver disease.

### Antibodies

Mouse anti-human IFI16 monoclonal antibody (sc-137,970) was obtained from Santa Cruz Biotechnology Inc., Santa Cruz, CA, USA. Kupffer cell (KC) marker rabbit anti-human CD68 (clone F7.2.38) polyclonal antibody, Natural killer (NK) cell marker rabbit anti-human CD56 (EP2567Y), liver sinusoidal endothelial cell (LESC) marker rabbit anti-human CD299 (EPR11211), dendritic cell (DC) marker rabbit anti-human CD11c (EP1347Y), and hepatic stellate cell (HSC) marker rabbit anti-human SMA (E184) monoclonal antibodies were purchased from ABclonal Biotech Co., Ltd. (Woburn, MA, USA) and Abcam’s RabMAb®technology (Cambridge, UK), respectively. Alexa Fluor® 488 Goat Anti-Mouse IgG and Alexa Fluor® 555 Goat Anti-Rabbit IgG antibodies were obtained from Life Technologies (Grand Island, NY,USA).

### PBMC isolation and flow cytometry

PBMCs were purified from EDTA-treated blood using a Ficoll density gradient. For intranuclear IFI16 staining, PBMCs (200 μL) were incubated in 800 μL of RPMI 1640 medium. The cells were treated with Human TruStain FcX, Nuclear Factor Fixation Buffer, and Nuclear Factor Permeabilization Buffer (Biolegend, San Diego, CA, USA) and stained with the IFI16 antibody and Alexa Fluor® 488 Goat Anti-Mouse IgG. Data collection was performed on a BDFACSCantoII (BD Biosciences, San Jose, CA, USA), and data files were analyzed using FlowJo software (FlowJo, LLC, Ashland, OR, USA). Statistical analysis was based on IFI16 staining of at least 10,000 gated cells.

### Liver pathology

The liver biopsy specimens were considered reliable when the liver specimen length was ≥1.5 cm. The liver biopsy specimens were fixed in formaldehyde, embedded in paraffin, cut into 4-μm sections, and conventionally stained with hematoxylin and eosin. Images were acquired using an Olympus Leica DM4000B microscope. According to the Scheuer scoring system, hepatic inflammation activity grade (G) was divided into G0 (no hepatic necroinflammation) and G1-G4 and hepatic fibrosis stage was divided into F0 (no hepatic fibrosis) and F1-F4. The degree of hepatic inflammation and fibrosis was graded using the modified histology activity index (HAI) [14] described by Scheuer. Liver specimens were interpreted by experienced liver pathologists. Sections of liver tissue were scored in a blinded fashion to assess their histological diagnoses.

### Immunohistochemistry

Immunohistochemistry was carried out using standard techniques [15]. The expression of IFI16-positive cells was determined by image analysis of the histological sections. Ten sections from each sample were randomly selected, and photomicrographs were obtained under high-power fields (0.625 mm^2^) and captured for analysis using Image Pro-Plus 6.0 software (Media Cybernetics, Silver Spring, MD, USA). The number of IFI16-positive cells per high-power field was counted and expressed as the mean + the standard error of the mean (SEM).

### Immunofluorescence double staining

For immunofluorescence double staining, paraffin-embedded tissue blocks were cut into 3.5-μm sections. After dewaxing and rehydration, antigen retrieval was performed with EDTA (pH 8.0). Then, sections were incubated at 4 °C overnight with primary anti-IFI16 (1:50) and cell type-specific marker antibodies anti-CD68 (1:100), anti-CD11c (1:50), anti-CD56 (1:50), anti-CD299 (1:100), and anti-SMA (1:1000). The sections were then incubated with Alexa Fluor® 488 Goat Anti-Mouse IgG and Alexa Fluor® 555 Goat Anti-Rabbit IgG Antibodies (1:1000) for 1 h. Finally, the sections were incubated with 1 μg/ml Hoechst 33,342 (Sigma-Aldrich, St. Louis, MO, USA) to stain the nuclei. Sections incubated with the PBS control primary antibodies and fluorescently labeled secondary antibodies were used as negative controls. The results were analyzed using fluorescence microscopy (Leica Corp.).

### Western blotting analysis

Cells were lysed in radioimmunoprecipitation assay (RIPA) buffer supplemented with protease inhibitor cocktail (Sigma-Aldrich), sonicated, and clarified by centrifugation. Equal amounts of protein were separated by SDS-PAGE and electrophoretically transferred to nitrocellulose membranes. Membranes were incubated with primary antibodies and secondary antibodies conjugated to horseradish peroxidase (KPL, Gaithersburg, MD, USA). Immunoreactive bands were visualized using an ECL western blotting substrate (Pierce Chemical, Rockford, IL, USA). Blots were scanned using FluorChemFC2 software with the AlphaImager system (Alpha Innotech Corp., San Leandro, CA, USA). Figures shown are representative of three or more experiments each.

### Statistical analysis

All datum were analyzed using the SPSS version 13.0 software (SPSS Inc., Chicago, IL, USA) or GraphPad Prism 6 (GraphPad, La Jolla, CA, USA) and summarized as the means and standard errors (SEs) or medians and range. Differences invariables were analyzed using ANOVA and Student’s t-tests (for normally distributed data) or the Kruskal–Wallis and Mann–Whitney U-tests (for non-normally distributed data), as appropriate. Correlation analysis was evaluated by the Spearman rank correlation test. A two-sided *P* value < 0.05 was considered statistically significant.

## Results

### Expression of IFI16 in PBMCs of HBV patients and healthy controls

**Fig. 1:**
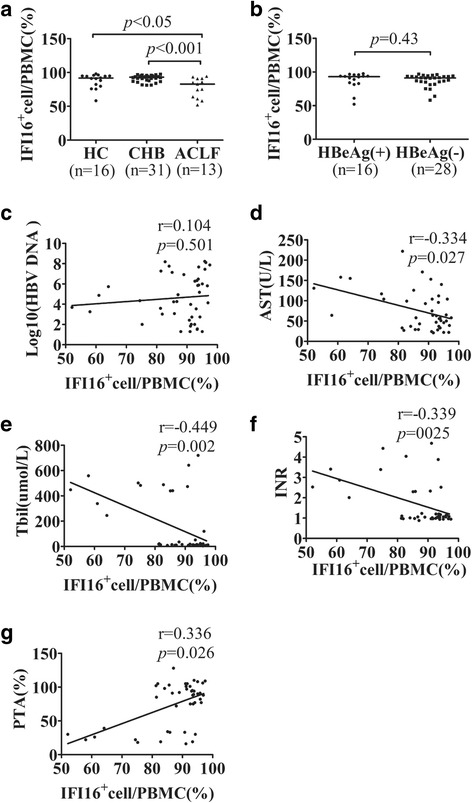
IFI16 expression levels in PBMCs from patients with ACLF, CHB, and healthy controls. There was a significant increase in IFI16 expression in ACLF patients compared with CHB patients and healthy controls (**a**). No significant difference was found between the HBeAg (+) and HBeAg (−) groups (**b**). No significant correlations were found between the IFI16 level and HBV DNA load (**c**). The expression of IFI16 was significantly negatively correlated with aspartate aminotransferase (AST) (**d**), serum total bilirubin (TBIL) (**e**), and the international normalized ratio(INR) (**f**), and positively correlated with prothrombin time activity (PTA) (**g**). AST aspartate aminotransferase, CHB chronic hepatitis B, ACLF acute-on-chronic liver failure, HBeAg hepatitis B e antigen, HBV hepatitis B virus, PBMCs peripheral blood mononuclear cells, TBIL total bilirubin, PTA prothrombin time activity, INR international normalized ratio

We first examined the expression of IFI16 in PBMCs from 31 patients with CHB,13 patients with ACLF, and 16 healthy controls using flow cytometry. The number of IFI16-positive cells in patients with ACLF was significantly lower than the number in patients with CHB and in healthy controls (median [25th percentile; 75th percentile], 82.80 [62.53, 90.95] vs 93.00 [87.95, 95.00], and 91.63 [87.95, 95.00], *P* < 0.001 and *P* < 0.05; Fig. 1a). There was no difference between CHB patients and healthy controls(*P* > 0.05). Then, we tested for a relationship between IFI16 expression levels and HBV parameters. There was no significant difference between the HBeAg(+) and HBeAg(−) groups (*P* = 0.43),and no statistically significant relationships were found between IFI16 expression levels and HBV DNA load (*r* = 0.104, *P* = 0.501; Fig. 1 b-c). In addition, we tested for a relationship between IFI16 expression levels and HBV DNA load only in chronic HBV infected patients, there was no statistically significant relationships (*r* = 0.335, *P* = 0.065). To further identify whether IFI16 expression contributed to the severity of CHB and ACLF, we evaluated the correlations between IFI16 and clinical parameters, including serum ALT, AST, TBIL, prothrombin time activity (PTA), and INR, in patients with HBV. IFI16 expression levels were positively correlated with PTA (*r* = 0.336, *P* = 0.026; Fig. 1g) and negatively correlated with AST (*r* = − 0.334, *P* = 0.027), INR (*r* = − 0.339, *P* = 0.025), and TBIL (*r* = − 0.449, *P* = 0.002) (Figs. 1d–f). No statistically significant relationships were found between IFI16 expression levels and ALT (*r* = − 0.027, *P* = 0.861). These results suggest the possibility that aberrant expression of IFI16 might be involved in the development of CHB and ACLF, especially in ACLF patients, and may be closely associated with disease severity.

### Expression of intrahepatic IFI16 protein in CHB and ACLF patients

**Fig. 2:**
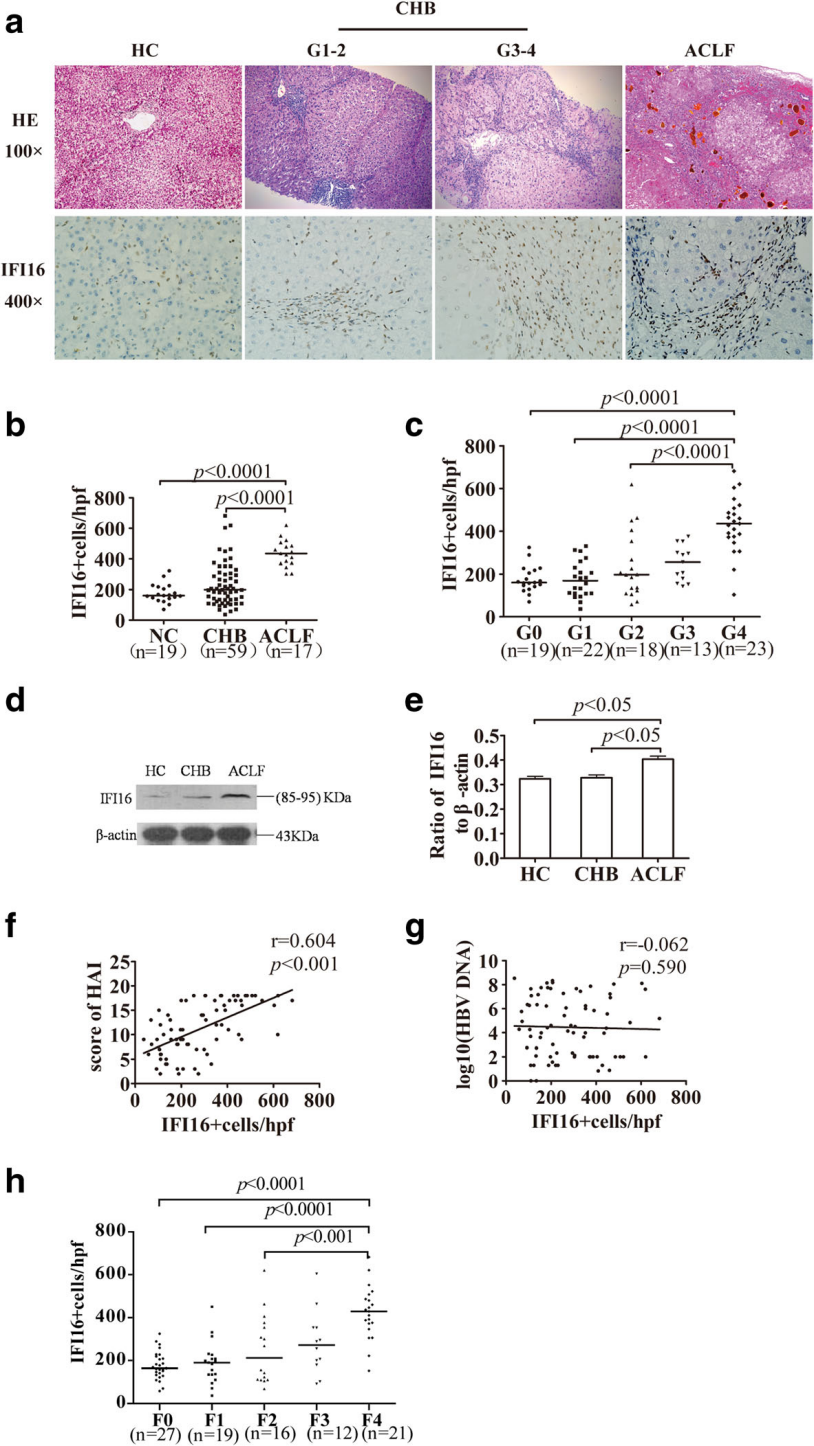
IFI16 expression levels in livers from patients with ACLF, CHB, and healthy controls. Different degrees of damage can be observed in the liver tissue from CHB and HBV-ACLF patients, as demonstrated by H&E staining. IFI16 expression was observed in the livers of healthy donors and patients with HBV and was mainly detected in liver non-parenchymal cells, especially the inflammatory cells in the livers of HBV patients(**a**). There was a significant increase in IFI16 expression in ACLF patients compared with CHB patients and healthy controls (**b**). In detail, the intrahepatic IFI16 expression in patients with inflammation grade 4 was significantly higher than those with inflammation grade 0, 1, 2, or 3 (**c**). The expression of IFI16 protein is shown by western blotting (**d**). The protein level of IFI16 in the HBV-ACLF group was remarkably higher than that of the CHB and normal control groups (**e**). The expression of IFI16 was significantly positively correlated with HAI (**f**). No significant correlations were found between the IFI16 level and HBV DNA load (**g**). The intrahepatic IFI16 expression in patients with fibrosis stage 4 was also significantly higher than in those with fibrosis stage 0,1, or 2 (**h**)

Furthermore, we evaluated the intrahepatic expression of IFI16 protein in 59 patients with CHB, 17 HBV-ACLF patients who received a liver transplant, and 19 healthy liver transplant donors. To measure the expression of IFI16 in the livers of CHB and ACLF patients, we used immunohistochemistry with anti-IFI16 to probe sections of paraffin-embedded livers. First, we saw different degrees of damage to the liver tissue from CHB and HBV-ACLF patients, as demonstrated by H&E staining (Fig. 2a). IFI16 expression that was observed in the livers of healthy donors and patients with HBV was mainly in non-parenchymal liver cells, especially the inflammatory cells in the livers of HBV patients. Furthermore, intrahepatic IFI16 expression gradually increased in accord with elevations in the grade of liver inflammation (Fig. 2a). Then, a semi-quantitative analysis of IFI16 expression in liver tissues was conducted using the software Image-Pro-Plus 6.0 to measure the number of IFI16-positive cells. The number of IFI16-positive cells in patients with ACLF was significantly higher than that in those with CHB or healthy controls (median [25th percentile; 75th percentile], 435.3 [372.9496.5] vs 199.6 [188.0, 307.1] vs160.8 [142.0, 218.0], both *P* < 0.0001; Fig. 2b). There was also no difference between the CHB patients and healthy controls (*P* > 0.05). In detail, the intrahepatic IFI16 expression in patients with inflammation grade 4 was significantly higher than in those with inflammation grade 0,1, or 2 (*P* < 0.0001; Fig. 2c). Moreover we found the intrahepatic IFI16 expression in patients with fibrosis stage 4 was also significantly higher than in those with fibrosis stage 0,1, or 2 (P < 0.0001; Fig. 2h). To further confirm our results obtained from the semi-quantitative immunohistochemistry, the protein level of IFI16 was analyzed by a western blotting analysis of cell extracts from liver tissues isolated from the different groups. As shown in Fig. 2d, the protein level of IFI16 in the HBV-ACLF group was remarkably higher than in the CHB(*P* = 0.006) and normal control groups (*P* = 0.001), and there is no difference between CHB and normal control groups (*P >* 0.05; Fig. 2e). In particular, this finding was consistent with the semi-quantitative immunohistochemistry levels for all groups. Then, we evaluated the correlations between IFI16 and clinical parameters, including HAI, serum AST, TBIL, PTA, and INR, in patients with HBV. IFI16 expression levels were positively correlated with HAI (*r* = 0.604, *P* < 0.001) (Fig. 2f), AST (*r* = 0.468, P < 0.001), INR (*r* = 0.562, P < 0.001), and TBIL (*r* = 0.515, P < 0.001) and negatively correlated with PTA (*r* = − 0.564, P < 0.001).We also found that there was no significant difference between the HBeAg (+) and HBeAg (−) groups (*P* = 0.773), and no statistically significant relationships were found between IFI16 expression levels and HBV DNA load (*r* = − 0.062, *P* = 0.590; Fig. 2g). Moreover, there was no statistically significant relationships were found between IFI16 expression levels and HBV DNA load only in chronic HBV infected patients (*r* = 0.169, *P* = 0.198).

### The distribution of IFI16 in common liver innate cells

**Fig. 3:**
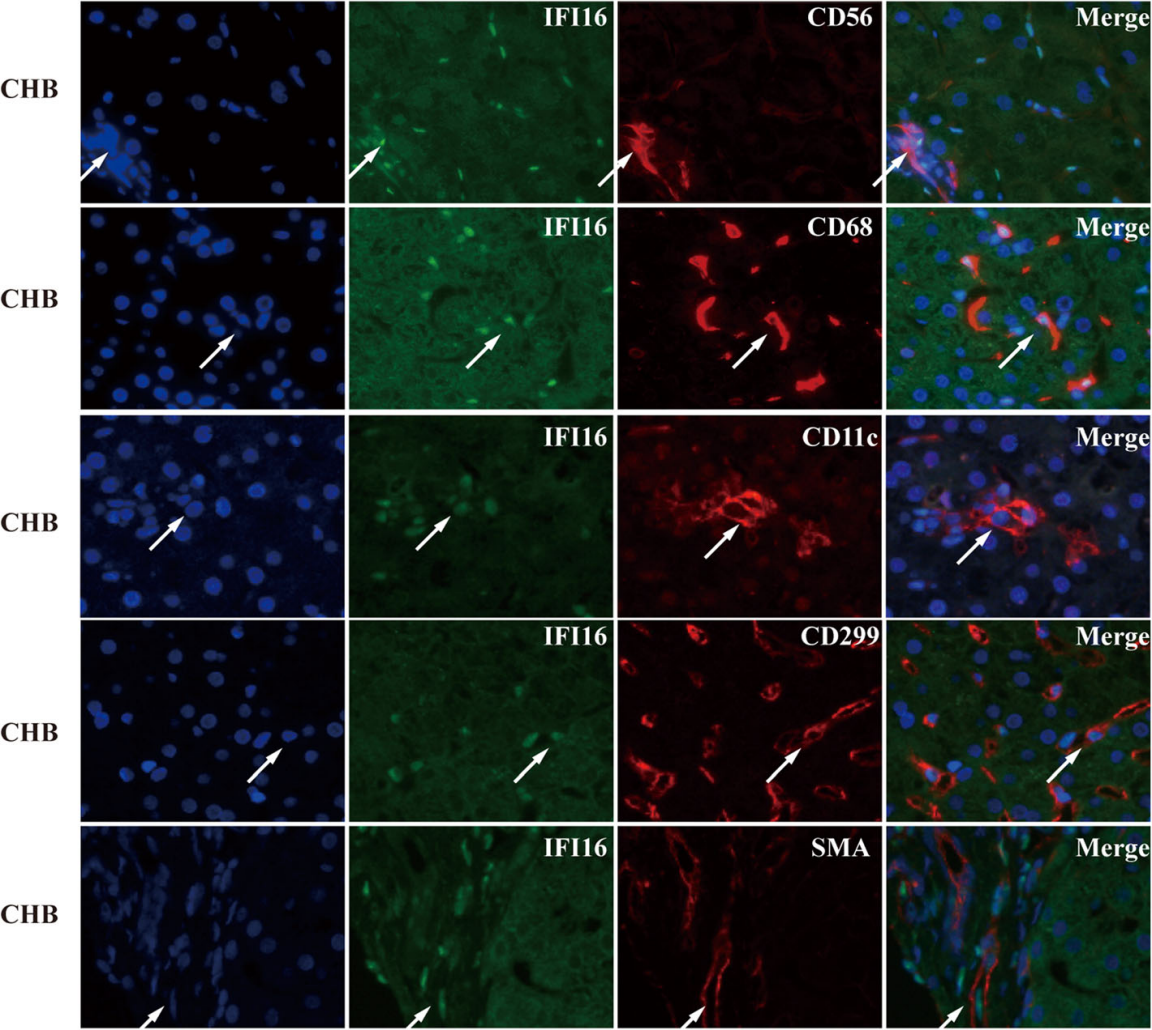
The morphology of IFI16^+^ cells in liver tissues from healthy donors and CHB patients was detected by immunofluorescence double staining. Immunofluorescence double staining revealed that IFI16 was expressed by CD299^+^ endothelial cells, CD68^+^ macrophages, CD56^+^ NK cells, CD11c^+^dendritic cells, and α-SMA^+^ hepatic stellate cells. Arrows indicate positive cells. The nuclei were stained with Hoechst33342, and the scale bar indicates 20 μm

**Fig. 4:**
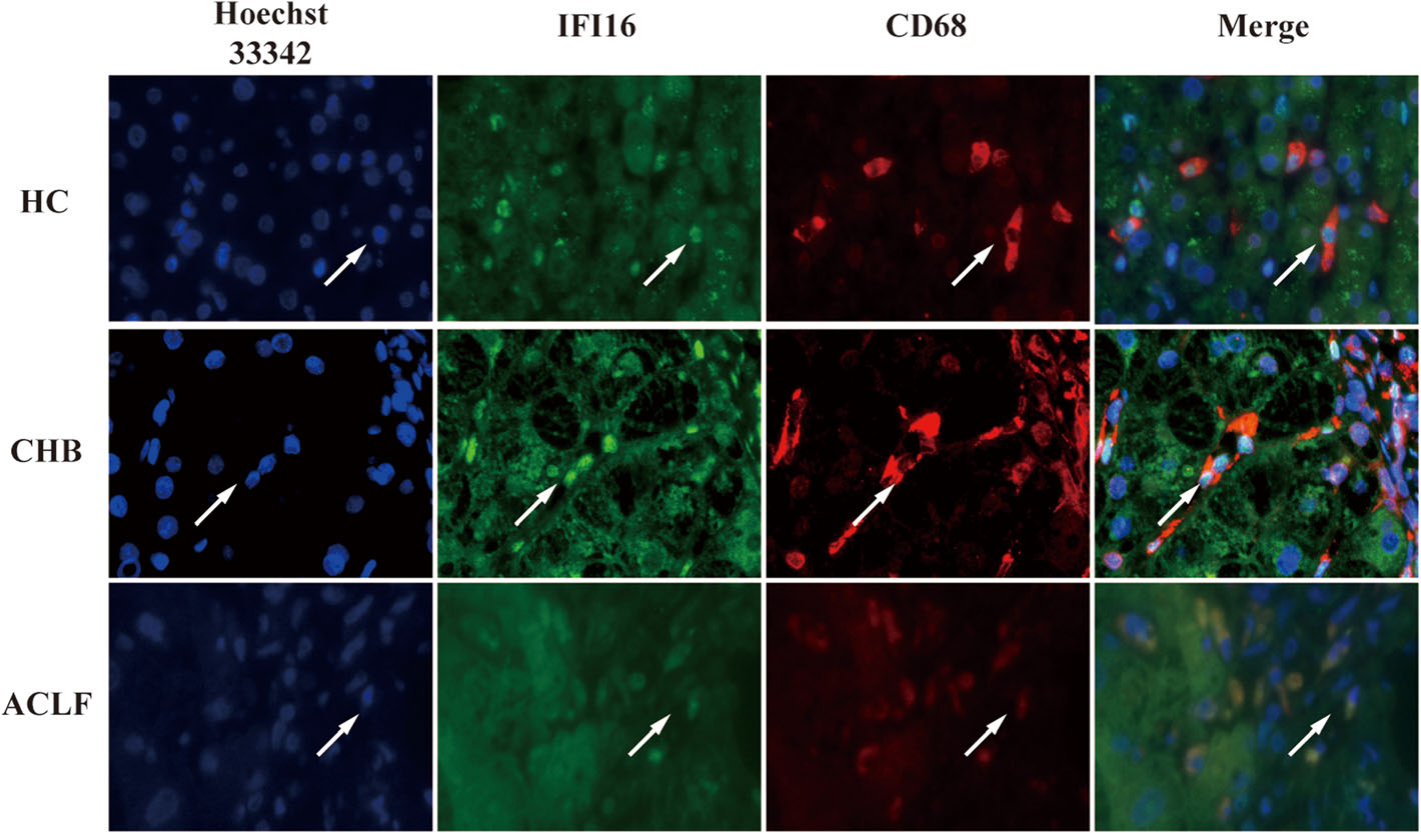
The localization of IFI16+ cells in CD68+ macrophages from the livers of healthy donors, CHB patients, and ACLF patients was detected by immunofluorescence double staining. Immunofluorescence double staining revealed that IFI16 was localized in the nucleus of CD68+ macrophages in healthy donors and CHB patients but in the cytoplasm of HBV-ACLF patients. Arrows indicate positive cells

The liver is well-known to be an immunological organ with a predominant innate immune system. The phenotypes of IFI16+ cells in liver tissues were further examined by immune fluorescence double staining. From these analyses, the expression of IFI16 in healthy donors and CHB patients was observed in the nucleus of CD299^+^ endothelial cells, CD68^+^KCs, CD56^+^ NK cells, CD11c^+^DCs, α-SMA^+^HSCs (Fig. 3), but this expression was completely absent from CD299^+^ endothelial cells, CD56^+^ NK cells, CD11c^+^DCs, and α-SMA^+^HSCs in HBV-ACLF patients. To our surprise, we found that IFI16 was expressed in the cytoplasm of CD68^+^KCs in HBV-ACLF patients (Fig. 4).

## Discussion

IFI16 was first reported in 1992, and subsequently research found its expression was associated with human myeloid cell differentiation [16]. Dawson and Trapani first discovered a DNA binding domain in IFI16 protein [17], and Leonie Unterholznerl identified IFI16 as an intracellular DNA sensor that mediates the induction of IFN [18]. Recent evidences show that IFI16 is involved in viral infection procedure and specifically serve as an inhibitor to viral infections. In addition, IFI16 mediates IFN-γ induction and stimulates inflammatory activation in certain cell types during infection with KSHV [8],HSV-1 [7] and HIV [19]. IFI16 may also act as a possible target in autoimmune diseases [20] and can be found in human fibroblasts, endothelial cells, and B cells. We hypothesized that IFI16 contributes to HBV infection and HBV-associated liver inflammation.

Here, we show for the first time that IFI16 expression levels are associated with liver inflammation in HBV-liver diseases. In particular, the number of IFI16+ PBMCs in HBV-ACLF patients was significantly lower than that in CHB patients and healthy controls. In contrast, the number of IFI16+ cells in livers of HBV-ACLF patients was significantly higher than that in CHB patients and healthy controls. In livers and PBMCs, there was no significant difference between CHB patients and healthy controls. Together, these results imply that the expression of IFI16 may predict the development of HBV-associated ACLF and that IFI16 may play an important role in disease pathogenesis. Though the precise mechanisms involved in HBV-associated ACLF have yet to be clarified, the host immune response and the inflammatory cascade are critical in this syndrome. The role that cytokines, including tumor necrosis factor-α (TNF-α), IL-1, and IFNs, play in HBV-ACLF remains a key unresolved point in our understanding of the pathogenesis of the inflammatory response. The rise in cytokine levels can be related to necrotic liver cells, decreased hepatic clearance, or the activation of PRRs [2]. Hyperactivation of the PRR signaling pathway is involved in liver injury [21]. IFI16 has now been identified as one of the PRRs. The receptor can sense DNA, then induce STING-dependent IFN expression and stimulate ASC-dependent inflammatory activation, such as the activation of caspase-1, which cleaves pro-IL-1β and pro-IL-18 to generate their active forms. Overexpression of IFI16 in the liver contributes to the injury, but the expression of IFI16 in PBMCs was lower in ACLF patients. We hypothesize that peripheral IFI16+ cells are selectively recruited to the liver, where they will mature and initiate a destructive immune response, leading to liver injury. This hypothesis will be tested in further studies.

IFI16 is categorized as one of the PYHIN family of proteins that are defined by an N-terminal PYD and one or two C-terminal DNA-binding HIN domains. According to its crystal structures, IFI16 can bind directly to different types of DNA via the C-terminal HIN-200 domain [18]. We speculated that IFI16 may sense HBV DNA to trigger the innate immune response, but our study did not uncover a statistically significant relationship between IFI16 expression levels and HBV parameters. In addition, there was no significant difference in the protein levels between CHB patients and healthy controls in livers or in PBMCs. This result suggests that HBV DNA cannot directly bind to IFI16. One possibility is that HBV DNA may have evolved mechanisms to counteract the actions of IFI16. Some studies have found that viruses have evolved mechanisms to counteract the actions of IFI16. For example, HSV-1 E3 ubiquitin ligase ICP0 targets IFI16 for degradation in human fibroblasts, thus impairing virus-induced expression of IFN-γ and IFN-stimulated genes [22].Human cytomegalovirus tegument protein pUL83 inhibits IFI16-mediated DNA sensing for immune evasion [11]. Another possibility is that IFI16 may only sense HBV DNA under special conditions. While DNA recognition by IFI16 can occur in both the nucleus and cytoplasm, acetylation at two different sites on the nuclear localization signal, situated in the N-terminal region of IFI16, has been recently discovered to regulate the nucleo-cytoplasmic localization of IFI16 [23].

Through further study, we identified the phenotypes of IFI16+ cells in liver tissues by immunofluorescence double staining. We found that IFI16 was localized in the nucleus of Kupffer cells (KCs), endothelial cells, natural killer (NK) cells, dendritic cells (DCs), and hepatic stellate cells(HSCs)in healthy donors and CHB patients, but in the liver of HBV-ACLF patients IFI16 was only localized in the cytoplasm of KCs. IFI16 was first described as a strictly nuclear protein, but more recent studies have indicated IFI16 can be found in the cytoplasm of many cell types [6]. For example, viral and DNA transfection modulates subcellular localization of IFI16 related to IFN induction and vesicle sorting pathways [23, 24]. Furthermore, UV–light treatment of epithelial cells driven IFI16 moving from the nucleus to back the cytoplasm [25]. This modulation of the localization of IFI16 is likely to impact the protein function. In this study, we found that IFI16 was only localized in the cytoplasm of KC which are cells that play a key role in liver injury by internalizing ligands, activating signaling cascades, and inducing transcription of proinflammatory cytokines and superoxide agents, thereby contributing to microcirculatory dysfunction. KCs are able to activate NK cells and natural killer T(NKT) cells, both of which are present at relatively high numbers in the liver, via the production of proinflammatory cytokines [26].In the future, we aim to understand how modulation of the subcellular localization of IFI16 in KCs contributes to the pathological mechanism of HBV-ACLF.

There are some limitations to the present study. First, we determined the IFI16 protein levels in only a small number of patients from a single unit. We cannot rule out the interference of age and believe that repeating the study in a community-based population or large cohort dataset would be helpful to understand the value of this marker. Second, other diseases that occur during the progression of HBV infection, including cirrhosis, should be included to explore the comprehensive role of IFI16 in the complete progression of HBV infection. However, it is hard to collect the samples from these patients. Actually, we found the intrahepatic IFI16 expression in patients of F4 was significantly higher than in F0 to F2. This is similar to what we found the relationship between IFI16 and inflammation. Researches show that chronic liver inflammation may lead to fibrosis and cirrhosis through IL-1signaling [27].And IFI16 can stimulate cleavage of pro-IL1b and pro-IL18 to the bioactive cytokines [6].So IFI16 may play a role in the pathogenesis of liver cirrhosis, next step we can further investigate it. Third, in future studies the effects of silencing the IFI16 gene in cell lines and HBV transgenic mice will be comprehensively investigated to understand the interplay between IFI16 and HBV.

## Conclusion

Our study revealed that the expression of IFI16 in HBV-ACLF patients was higher in livers but lower in PBMCs and that IFI16 was only localized in the cytoplasm of KCs. These results suggest that IFI16 might contribute to the pathogenesis of HBV-associated ACLF; however, the function of IFI16 during HBV infection remains to be determined. Further work is needed to elucidate the role of IFI16 in the liver and the importance of its subcellular localization in KCs.

## Abbreviations

AIM-2: absent in melanoma 2
ALT: alanine amino transferase
ASC: apoptosis-associated speck-like protein containing a caspase recruitment domain
AST: aspartate aminotransferase
CHB: chronic hepatitis B
DC: dendritic cell
EBV: Epstein–Barr virus
HBV-ACLF: HBV-associated acute-on-chronic liver failure
HCMV: human cytomegalovirus
HSC: hepatic stellate cell
HSV-1: herpes simplex virus 1
IBD: inflammatory bowel disease
IFI16: IFN inducible protein 16
IL-1: interleukin-1
ISGs: IFN-stimulated genes
KC: Kupffer cell
KSHV: Kaposi’s sarcoma-related herpesvirus
LESC: liver sinusoidal endothelial cell
MELD: Model for end-stage liver disease
NK: Natural Killer
NKT: natural killer T.
NLRs: NOD-like receptors
NLS: nuclear localization signal
PBMCs: peripheral blood mononuclear cells
PRRs: pattern-recognition receptors
PTA: prothrombin time activity
PYD: pyrin domain
RLRs: RIG-I-like receptors
STING: stimulate stimulator of IFN genes
Tbil: total bilirubin
TLRs: Toll-like receptors
TNF-α: tumor necrosis factor-α
